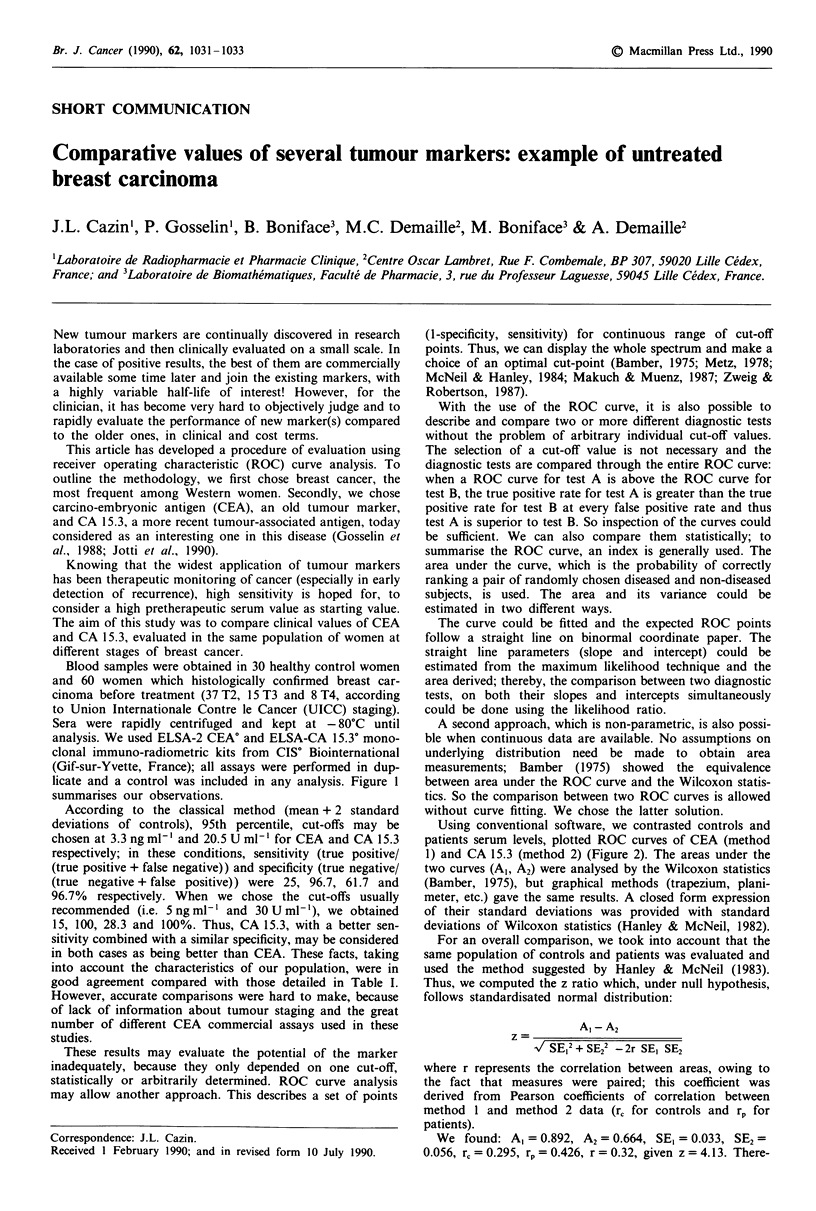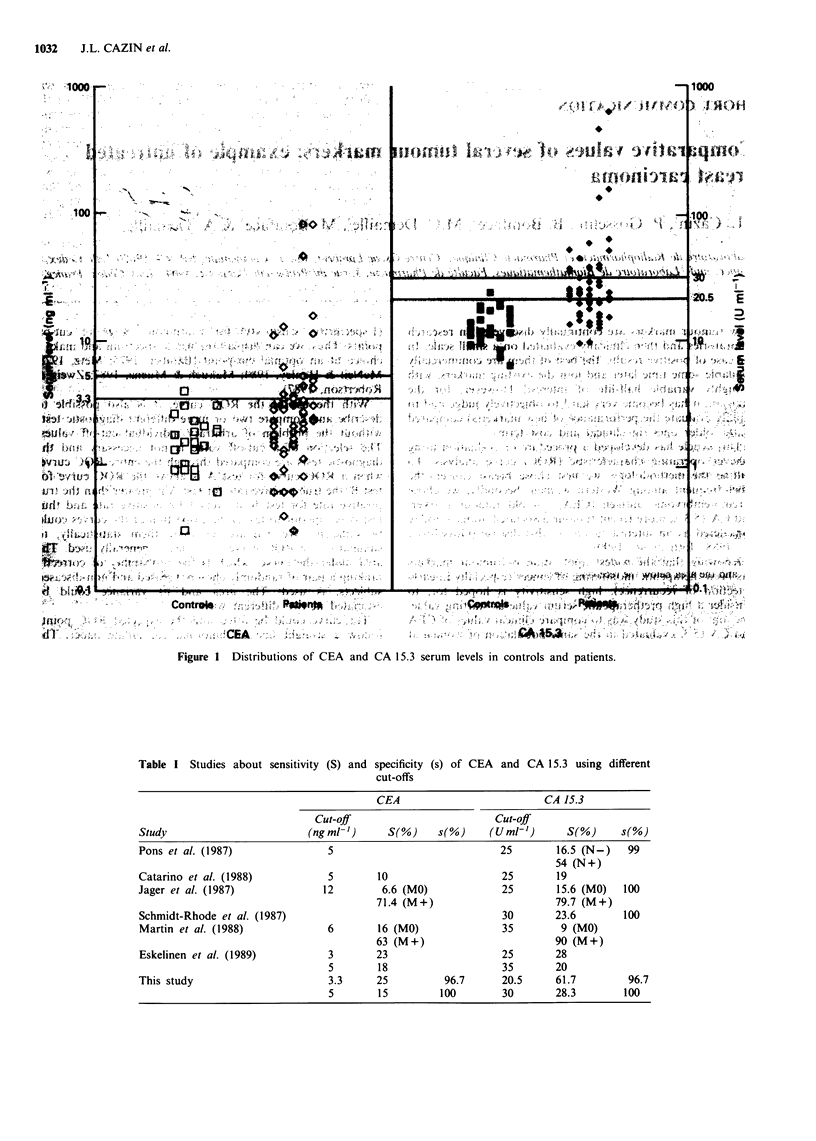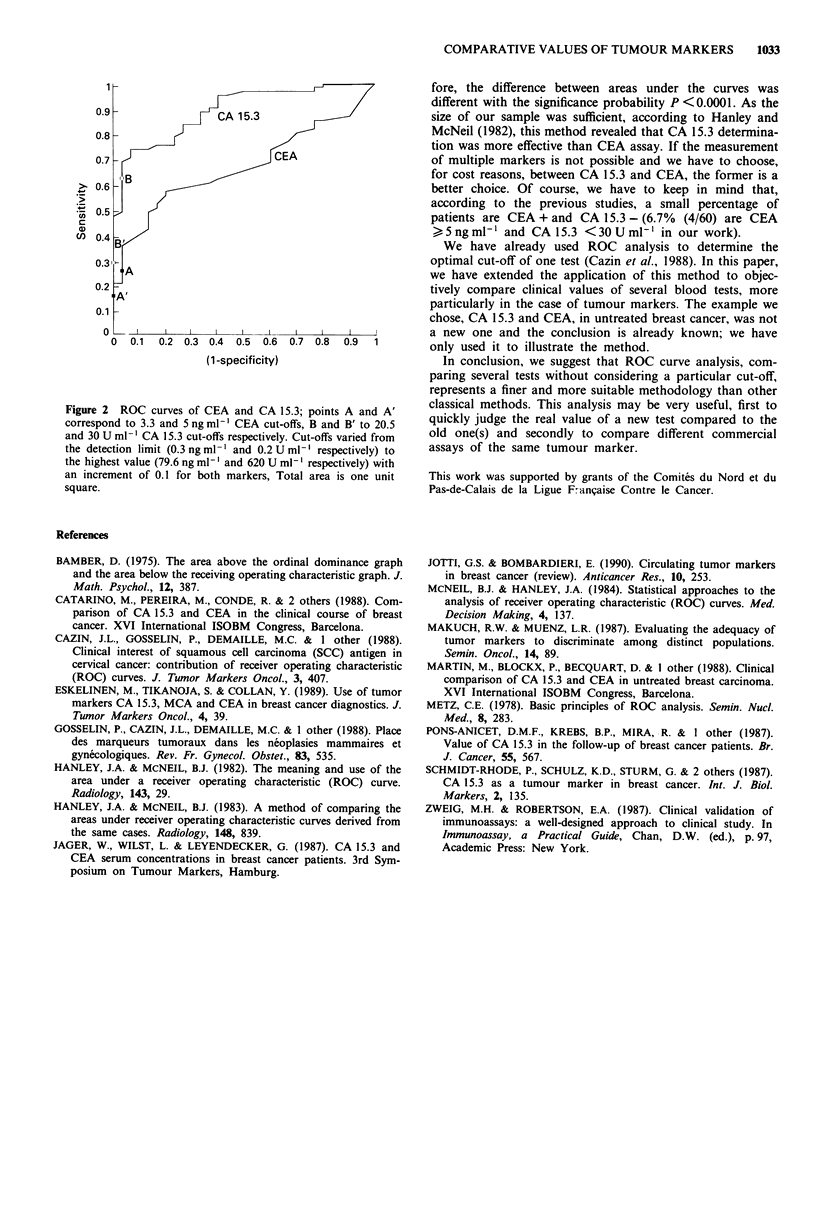# Comparative values of several tumour markers: example of untreated breast carcinoma.

**DOI:** 10.1038/bjc.1990.433

**Published:** 1990-12

**Authors:** J. L. Cazin, P. Gosselin, B. Boniface, M. C. Demaille, M. Boniface, A. Demaille

**Affiliations:** Laboratoire de Radiopharmacie et Pharmacie Clinique, Lille, France.


Br. J. Cancer (1990), 62, 1031-1033 © Macmillan Press Ltd., 1990

SHORT COMMUNICATION

### Comparative values of several tumour markers: example of untreated breast carcinoma

J.L. Cazin[1], P. Gosselin[1], B. Boniface[3], M.C. Demaille[2], M. Boniface[3] & A. Demaille[2]

[1]*Laboratoire de Radiopharmacie et Pharmacie Clinique,* [2]*Centre Oscar Lambret, Rue F. Combemale, BP 307, 59020 Lille Cédex, France; and* [3]*Laboratoire de Biomathématiques, Faculté de Pharmacie, 3, rue du Professeur Laguesse, 59045 Lille Cédex, France.*

New tumour markers are continually discovered in research laboratories and then clinically evaluated on a small scale. In the case of positive results, the best of them are commercially available some time later and join the existing markers, with a highly variable half-life of interest! However, for the clinician, it has become very hard to objectively judge and to rapidly evaluate the performance of new marker(s) compared to the older ones, in clinical and cost terms.

This article has developed a procedure of evaluation using receiver operating characteristic (ROC) curve analysis. To outline the methodology, we first chose breast cancer, the most frequent among Western women. Secondly, we chose carcino-embryonic antigen (CEA), an old tumour marker, and CA 15.3, a more recent tumour-associated antigen, today considered as an interesting one in this disease (Gosselin *et al.*, 1988; Jotti *et al.*, 1990).

Knowing that the widest application of tumour markers has been therapeutic monitoring of cancer (especially in early detection of recurrence), high sensitivity is hoped for, to consider a high pretherapeutic serum value as starting value. The aim of this study was to compare clinical values of CEA and CA 15.3, evaluated in the same population of women at different stages of breast cancer.

Blood samples were obtained in 30 healthy control women and 60 women which histologically confirmed breast carcinoma before treatment (37 T2, 15 T3 and 8 T4, according to Union Internationale Contre le Cancer (UICC) staging). Sera were rapidly centrifuged and kept at −80°C until analysis. We used ELSA-2 CEA° and ELSA-CA 15.3° monoclonal immuno-radiometric kits from CIS° Biointernational (Gif-sur-Yvette, France); all assays were performed in duplicate and a control was included in any analysis. Figure 1 summarises our observations.

According to the classical method (mean + 2 standard deviations of controls), 95th percentile, cut-offs may be chosen at 3.3 $ng\ ml^{-1}$ and 20.5 $U\ ml^{-1}$ for CEA and CA 15.3 respectively; in these conditions, sensitivity (true positive/(true positive + false negative)) and specificity (true negative/(true negative + false positive)) were 25, 96.7, 61.7 and 96.7% respectively. When we chose the cut-offs usually recommended (i.e. 5 $ng\ ml^{-1}$ and 30 $U\ ml^{-1}$), we obtained 15, 100, 28.3 and 100%. Thus, CA 15.3, with a better sensitivity combined with a similar specificity, may be considered in both cases as being better than CEA. These facts, taking into account the characteristics of our population, were in good agreement compared with those detailed in Table I. However, accurate comparisons were hard to make, because of lack of information about tumour staging and the great number of different CEA commercial assays used in these studies.

These results may evaluate the potential of the marker inadequately, because they only depended on one cut-off, statistically or arbitrarily determined. ROC curve analysis may allow another approach. This describes a set of points (1-specificity, sensitivity) for continuous range of cut-off points. Thus, we can display the whole spectrum and make a choice of an optimal cut-point (Bamber, 1975; Metz, 1978; McNeil & Hanley, 1984; Makuch & Muenz, 1987; Zweig & Robertson, 1987).

With the use of the ROC curve, it is also possible to describe and compare two or more different diagnostic tests without the problem of arbitrary individual cut-off values. The selection of a cut-off value is not necessary and the diagnostic tests are compared through the entire ROC curve: when a ROC curve for test A is above the ROC curve for test B, the true positive rate for test A is greater than the true positive rate for test B at every false positive rate and thus test A is superior to test B. So inspection of the curves could be sufficient. We can also compare them statistically; to summarise the ROC curve, an index is generally used. The area under the curve, which is the probability of correctly ranking a pair of randomly chosen diseased and non-diseased subjects, is used. The area and its variance could be estimated in two different ways.

The curve could be fitted and the expected ROC points follow a straight line on binormal coordinate paper. The straight line parameters (slope and intercept) could be estimated from the maximum likelihood technique and the area derived; thereby, the comparison between two diagnostic tests, on both their slopes and intercepts simultaneously could be done using the likelihood ratio.

A second approach, which is non-parametric, is also possible when continuous data are available. No assumptions on underlying distribution need be made to obtain area measurements; Bamber (1975) showed the equivalence between area under the ROC curve and the Wilcoxon statistics. So the comparison between two ROC curves is allowed without curve fitting. We chose the latter solution.

Using conventional software, we contrasted controls and patients serum levels, plotted ROC curves of CEA (method 1) and CA 15.3 (method 2) (Figure 2). The areas under the two curves ($A_1$, $A_2$) were analysed by the Wilcoxon statistics (Bamber, 1975), but graphical methods (trapezium, planimeter, etc.) gave the same results. A closed form expression of their standard deviations was provided with standard deviations of Wilcoxon statistics (Hanley & McNeil, 1982).

For an overall comparison, we took into account that the same population of controls and patients was evaluated and used the method suggested by Hanley & McNeil (1983). Thus, we computed the z ratio which, under null hypothesis, follows standardisated normal distribution:

$$z = \frac{A_1 - A_2}{\sqrt{SE_1^2 + SE_2^2 - 2r\ SE_1\ SE_2}}$$

where r represents the correlation between areas, owing to the fact that measures were paired; this coefficient was derived from Pearson coefficients of correlation between method 1 and method 2 data ($r_c$ for controls and $r_p$ for patients).

We found: $A_1 = 0.892$, $A_2 = 0.664$, $SE_1 = 0.033$, $SE_2 = 0.056$, $r_c = 0.295$, $r_p = 0.426$, $r = 0.32$, given $z = 4.13$. There-

Correspondence: J.L. Cazin.
Received 1 February 1990; and in revised form 10 July 1990.

**Figure 1** Distributions of CEA and CA 15.3 serum levels in controls and patients.

**Table I** Studies about sensitivity (S) and specificity (s) of CEA and CA 15.3 using different cut-offs

| | CEA | | | CA 15.3 | | |
|---|---|---|---|---|---|---|
| *Study* | *Cut-off (ng ml$^{-1}$)* | *S(%)* | *s(%)* | *Cut-off (U ml$^{-1}$)* | *S(%)* | *s(%)* |
| Pons *et al.* (1987) | 5 | | | 25 | 16.5 (N−)<br>54 (N+) | 99 |
| Catarino *et al.* (1988) | 5 | 10 | | 25 | 19 | |
| Jager *et al.* (1987) | 12 | 6.6 (M0)<br>71.4 (M+) | | 25 | 15.6 (M0)<br>79.7 (M+) | 100 |
| Schmidt-Rhode *et al.* (1987) | | | | 30 | 23.6 | 100 |
| Martin *et al.* (1988) | 6 | 16 (M0)<br>63 (M+) | | 35 | 9 (M0)<br>90 (M+) | |
| Eskelinen *et al.* (1989) | 3 | 23 | | 25 | 28 | |
| | 5 | 18 | | 35 | 20 | |
| This study | 3.3 | 25 | 96.7 | 20.5 | 61.7 | 96.7 |
| | 5 | 15 | 100 | 30 | 28.3 | 100 |

**Figure 2** ROC curves of CEA and CA 15.3; points A and A' correspond to 3.3 and 5 ng ml$^{-1}$ CEA cut-offs, B and B' to 20.5 and 30 U ml$^{-1}$ CA 15.3 cut-offs respectively. Cut-offs varied from the detection limit (0.3 ng ml$^{-1}$ and 0.2 U ml$^{-1}$ respectively) to the highest value (79.6 ng ml$^{-1}$ and 620 U ml$^{-1}$ respectively) with an increment of 0.1 for both markers, Total area is one unit square.

fore, the difference between areas under the curves was different with the significance probability $P < 0.0001$. As the size of our sample was sufficient, according to Hanley and McNeil (1982), this method revealed that CA 15.3 determination was more effective than CEA assay. If the measurement of multiple markers is not possible and we have to choose, for cost reasons, between CA 15.3 and CEA, the former is a better choice. Of course, we have to keep in mind that, according to the previous studies, a small percentage of patients are CEA + and CA 15.3 − (6.7% (4/60) are CEA $\geqslant$5 ng ml$^{-1}$ and CA 15.3 $<$30 U ml$^{-1}$ in our work).

We have already used ROC analysis to determine the optimal cut-off of one test (Cazin *et al.*, 1988). In this paper, we have extended the application of this method to objectively compare clinical values of several blood tests, more particularly in the case of tumour markers. The example we chose, CA 15.3 and CEA, in untreated breast cancer, was not a new one and the conclusion is already known; we have only used it to illustrate the method.

In conclusion, we suggest that ROC curve analysis, comparing several tests without considering a particular cut-off, represents a finer and more suitable methodology than other classical methods. This analysis may be very useful, first to quickly judge the real value of a new test compared to the old one(s) and secondly to compare different commercial assays of the same tumour marker.

This work was supported by grants of the Comités du Nord et du Pas-de-Calais de la Ligue Française Contre le Cancer.

### References

BAMBER, D. (1975). The area above the ordinal dominance graph and the area below the receiving operating characteristic graph. *J. Math. Psychol.*, **12**, 387.

CATARINO, M., PEREIRA, M., CONDE, R. & 2 others (1988). Comparison of CA 15.3 and CEA in the clinical course of breast cancer. XVI International ISOBM Congress, Barcelona.

CAZIN, J.L., GOSSELIN, P., DEMAILLE, M.C. & 1 other (1988). Clinical interest of squamous cell carcinoma (SCC) antigen in cervical cancer: contribution of receiver operating characteristic (ROC) curves. *J. Tumor Markers Oncol.*, **3**, 407.

ESKELINEN, M., TIKANOJA, S. & COLLAN, Y. (1989). Use of tumor markers CA 15.3, MCA and CEA in breast cancer diagnostics. *J. Tumor Markers Oncol.*, **4**, 39.

GOSSELIN, P., CAZIN, J.L., DEMAILLE, M.C. & 1 other (1988). Place des marqueurs tumoraux dans les néoplasies mammaires et gynécologiques. *Rev. Fr. Gynecol. Obstet.*, **83**, 535.

HANLEY, J.A. & MCNEIL, B.J. (1982). The meaning and use of the area under a receiver operating characteristic (ROC) curve. *Radiology*, **143**, 29.

HANLEY, J.A. & MCNEIL, B.J. (1983). A method of comparing the areas under receiver operating characteristic curves derived from the same cases. *Radiology*, **148**, 839.

JAGER, W., WILST, L. & LEYENDECKER, G. (1987). CA 15.3 and CEA serum concentrations in breast cancer patients. 3rd Symposium on Tumour Markers, Hamburg.

JOTTI, G.S. & BOMBARDIERI, E. (1990). Circulating tumor markers in breast cancer (review). *Anticancer Res.*, **10**, 253.

MCNEIL, B.J. & HANLEY, J.A. (1984). Statistical approaches to the analysis of receiver operating characteristic (ROC) curves. *Med. Decision Making*, **4**, 137.

MAKUCH, R.W. & MUENZ, L.R. (1987). Evaluating the adequacy of tumor markers to discriminate among distinct populations. *Semin. Oncol.*, **14**, 89.

MARTIN, M., BLOCKX, P., BECQUART, D. & 1 other (1988). Clinical comparison of CA 15.3 and CEA in untreated breast carcinoma. XVI International ISOBM Congress, Barcelona.

METZ, C.E. (1978). Basic principles of ROC analysis. *Semin. Nucl. Med.*, **8**, 283.

PONS-ANICET, D.M.F., KREBS, B.P., MIRA, R. & 1 other (1987). Value of CA 15.3 in the follow-up of breast cancer patients. *Br. J. Cancer*, **55**, 567.

SCHMIDT-RHODE, P., SCHULZ, K.D., STURM, G. & 2 others (1987). CA 15.3 as a tumour marker in breast cancer. *Int. J. Biol. Markers*, **2**, 135.

ZWEIG, M.H. & ROBERTSON, E.A. (1987). Clinical validation of immunoassays: a well-designed approach to clinical study. In *Immunoassay, a Practical Guide*, Chan, D.W. (ed.), p. 97, Academic Press: New York.